# TikTok as a Source of Medical Information: Port Wine Stain Birthmarks and Treatment Using Vascular Lasers

**DOI:** 10.7759/cureus.45119

**Published:** 2023-09-12

**Authors:** Hannah Tolson, Danielle Wenger, Mariana McCune, Fatmah Alzahrani, Elizabeth Dupuy

**Affiliations:** 1 Dermatology, University of Arizona College of Medicine, Phoenix, USA; 2 Medicine, University of Arizona College of Medicine, Phoenix, USA; 3 Pediatric Dermatology, Phoenix Children's Hospital, Phoenix, USA

**Keywords:** tiktok, healthcare technology, social media, healthcare delivery, dermatology

## Abstract

Background: As social media usage grows, more patients are turning to various platforms to gain and share medical information. One platform, TikTok, has become immensely popular, with over one billion users. Despite its potential use as an educational tool, TikTok can be unreliable and misleading as a medical information source.

Objective: We aim to discuss the information available on TikTok regarding laser treatment for port wine stains (PWS).

Methods: Two independent reviewers analyzed 200 TikTok videos with the hashtags #portwinestainlaser or #portwinestaintreatment, examining the video creator’s role (e.g., patient, parent, physician, or other), tone towards PWS and treatment options, and content (e.g., educational or non-educational, mention of any treatment risks).

Results:* *Most videos were produced by non-medical professionals (83%), and only a small number discussed the potential adverse effects of PWS treatment options (15%).

Conclusion: While TikTok may have a role in educating patients about certain dermatologic conditions and treatment options, it is important to encourage patients to seek medical advice from a qualified medical professional before making any treatment decisions. Furthermore, the future of patient education may need to evolve to include social media platforms.

## Introduction

The Internet is an increasingly popular resource for healthcare consumers. TikTok, the fastest-growing video-sharing social media network since 2019, represents a burgeoning platform for different groups of people to interface. Among TikTok videos that dispense medical information, dermatology-related TikTok videos are some of the most common [1]. This is likely due to the unique visual nature of dermatologic conditions, making them an ideal subject matter for this video platform [2]. 

Port wine stains (PWS) are congenital vascular malformations characterized by ectatic capillaries in the papillary layer of the dermis. They usually appear at birth, tend to become darker and thicker with age, and may be associated with other syndromes such as Sturge-Weber syndrome [3,4]. Pulse dye laser (PDL) is the gold standard for all PWS, regardless of lesion size, location, or color. Treatment of PWS is indicated to minimize psychosocial impact, diminish nodularity, and reduce tissue hypertrophy [5]. Videos providing advice about laser treatment are common on TikTok. 

Concerns exist about the correctness and completeness of medical advice available on TikTok. A study by Villa-Ruiz et al. characterized the content, sources, and reliability of the most popular dermatology videos on TikTok [6]. Using this study as a guide, we seek to describe the information about laser treatment for PWS birthmarks available on the platform. This article was previously presented as a meeting abstract at the 2023 ASLMS Annual Meeting on April 15, 2023.

## Materials and methods

Two independent reviewers conducted two searches using new, algorithm-naïve TikTok accounts with the hashtags #portwinestainlaser or #portwinestaintreatment on the same designated day. Each reviewer documented the first 100 videos discovered under each respective search and reviewed a total of 200 videos. These videos had the highest engagement for each tag, a TikTok metric based on the number of likes, comments, and shares. Promotional videos and videos that did not discuss PWS were excluded. Reviewers cross-referenced their recordings with each other to prevent redundancy. 

Documented data points included the video creator’s name, title (patient, medical doctors (MD) / doctors of osteopathic medicine (DO), parent, or other), presence of educational points, terms defined, tone toward PWS and its treatment, mention of laser treatment, and risks of treatment mentioned. Videos were said to include the definition of PWS if they mentioned the term "congenital birthmark" or an equivalent. Videos were said to have a positive tone if positive language was used (i.e., words like "excited", "thrilled", and "happy") or if nonverbal cues reflected a positive attitude as determined by the video reviewer. Likewise, videos were said to have a negative tone either if negative language was used (i.e., "sad," "scared," "upset") or if nonverbal cues reflected a negative tone. All other videos were considered tone-neutral.

A video was considered to have an educational point with regards to PWS if it possessed the following features: mention of the terms "vascular lesion", "blood vessel lesion", or an equivalent, or if it discussed a syndrome, a congenital nature, used the term birthmark, or discussed the development of the lesion. Videos were also considered educational if they discussed pathogenesis, such as the mention of sequelae of the disease, including chronic skin changes or syndromes associated with PWS, such as Sturge-Weber syndrome. 

## Results

**Figure 1:**
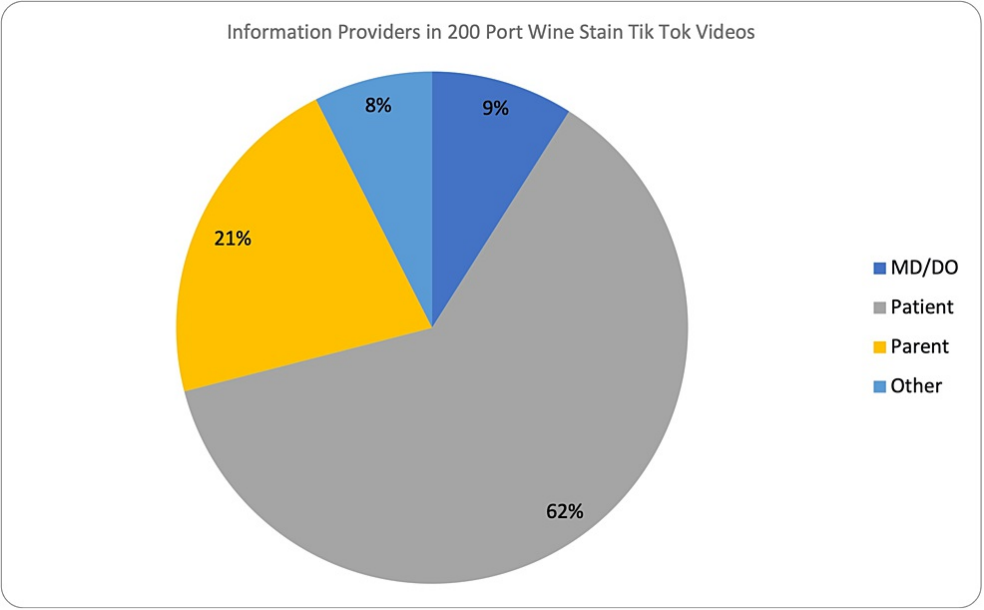
Pie chart demonstrating the distribution of information providers in 200 TikTok videos MD: Medical doctors, DO: Doctors of osteopathic medicine

Our results showed that of the 200 videos analyzed, 62% were recorded by patients, 21% by parents, 9% by medical doctors, and 8% by other healthcare professionals (Figure 1).

**Figure 2:**
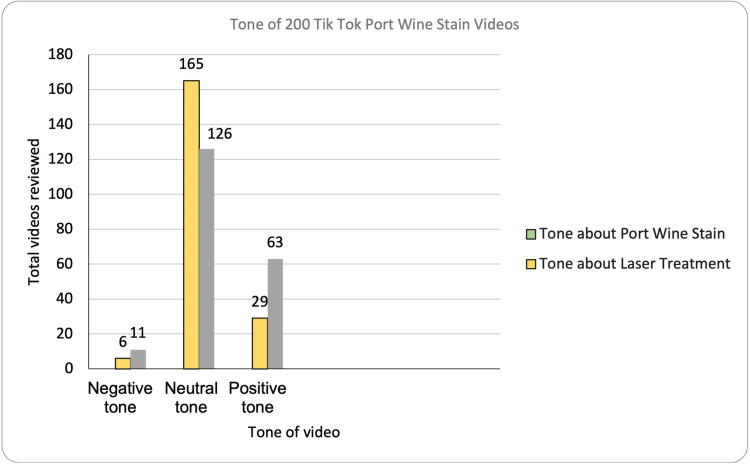
Bar chart demonstrating tone towards port wine stain (grey) and tone towards laser therapy (yellow) Raw total is noted above each bar with percentages calculated independently.

A total of 53% of videos contained at least one educational point, while 47% contained zero. The tone was overwhelmingly neutral in the videos analyzed, with 82% of videos taking a neutral tone when discussing PWS and 63% taking a neutral tone when discussing laser treatment (Figure 2).

**Figure 3:**
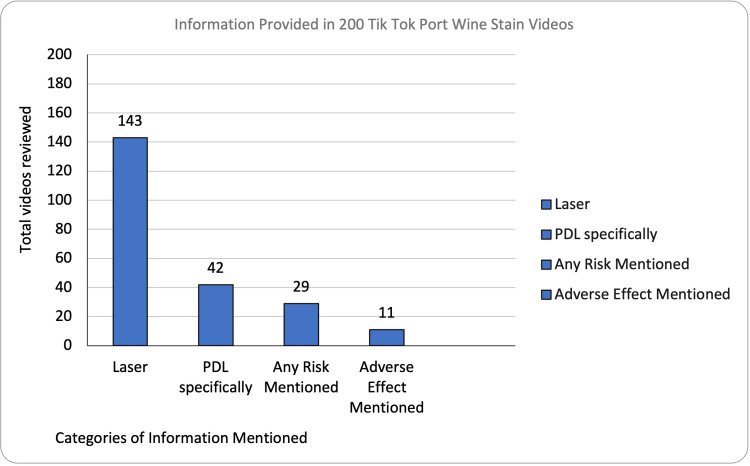
Bar chart demonstrating the content mentioned in each TikTok video Raw total is noted above each bar with percentages calculated independently. PDL: Pulse dye laser

Risks, adverse events, and safety concerns were only discussed in 15% of TikTok videos (Figure 3).

Several content creators appeared with significant frequency: 39% of all videos were produced by only four TikTok users.

## Discussion

Our results demonstrate that popular social media platforms, such as TikTok, have the potential to influence and inform the public about various dermatologic conditions like PWS. We found that the vast majority of TikTok videos were created by non-medical professionals (83%) and popular content creators (39% of all videos were produced by only four TikTok creators). We found that only a handful of the TikTok videos reviewed mentioned the possible adverse effects of laser treatment, and none of the videos mentioned safety concerns. A majority of videos discussing PWS and laser treatment options adopted a neutral tone rather than a negative or positive tone. Patients actively seeking PWS treatment must engage with their physician in a shared decision-making process to discuss the best treatment options for them.

One of our study’s main limitations is that we only looked at a limited number of TikTok videos; our subject matter specified PWS, and the selected videos may not have been the most representative of TikTok’s content related to PWS. Despite employing a dual independent reviewer strategy to minimize bias, there remained some element of subjectivity in the evaluation of video tone and content. We did not differentiate between patient anecdotes and direct advice given to viewers, which is important when considering medical misinformation. We also did not look at population data that could correlate with an increased number of consumers taking advice directly from TikTok. This could be a valuable future study to better quantify the effects that TikTok has on the dermatologic health of its users. 

Several other studies have also investigated the role of this popular platform in the dispersal of misinformation. Zheng et al. found that many dermatology-related TikTok videos share incorrect and potentially harmful medical information with the public [7]. A few of the dangerous at-home treatments for dermatologic conditions that have gained virality on the platform include at-home micro-needling, hyaluronic acid injections, replacing shampoo with olive oil, sunscreen contouring, and rubbing lemon and ice on the face [8]. Our study confirms that only a fraction of dermatology-related TikTok videos are created by dermatologists, as we found that only 9% of videos related to PWS and PWS treatment options were made by dermatologists. Researchers report there is a definite need to enhance the representation of board-certified dermatologists on the platform to generate more trustworthy and reliable medical material [3]. Additionally, there is a recommendation to integrate more "viral" components in these videos so that the content reaches a larger audience [7]. One article determined that the most common components of viral dermatology videos are music, healthcare attire, and on-screen text [9]. Future efforts to increase the accuracy of information on TikTok may include linking scientifically relevant articles and websites to video posts. Additionally, encouraging viewers to seek advice from healthcare professionals will mitigate many of the concerns surrounding TikTok information.

In 2021, Roche et al. analyzed the dermatology-related content on TikTok and quantified the view count of specific dermatological conditions on the platform. The study’s results found that #acne was the most popular search term with over 4.5 billion viewers [2,10]. TikTok has a relatively young audience, with 63.5% of users less than 24 years old [8]. This means there is an opportunity for board-certified dermatologists to use the platform to educate the younger generation. One study suggested linking peer-reviewed articles and evidence-based resources, requesting account verification to provide greater credibility, declaring conflicts of interest, and initiating targeted campaigns to help educate and dispel misinformation [6]. Research has shown that TikTok videos have the potential to influence the primary prevention of common dermatologic conditions [11]. Further research is needed to comprehensively understand the prevalence of dermatologic misinformation on social media platforms such as TikTok as well as the potential impact it may have on the health of patients. 

## Conclusions

TikTok remains an efficient and popular way to discuss treatment for dermatologic conditions. The majority (83%) of videos discussing the treatment of PWS with lasers were published by patients and parents, while only a minority (17%) were created by medical professionals. Our review revealed that there is little information about risks, adverse events, and safety concerns on TikTok. Since this platform is a popular source of information for patients and their families about PWS and laser treatment, physicians should engage with patients to determine what they understand from these videos to better fill in knowledge gaps.
